# Genome Sequences of Shewanella baltica and Shewanella morhuae Strains Isolated from the Gastrointestinal Tract of Freshwater Fish

**DOI:** 10.1128/genomeA.00541-18

**Published:** 2018-06-21

**Authors:** Daniel Castillo, Lone Gram, Frank E. Dailey

**Affiliations:** a Marine Biological Section, University of Copenhagen, Helsingør, Denmark; b Department of Biotechnology and Biomedicine, Technical University of Denmark, Kgs Lyngby, Denmark; c Department of Biology, University of Wisconsin–Sheboygan, Sheboygan, Wisconsin, USA

## Abstract

We present here the genome sequences of Shewanella baltica strain CW2 and Shewanella morhuae strain CW7, isolated from the gastrointestinal tract of Salvelinus namaycush (lean lake trout) and Coregonus clupeaformis (whitefish), respectively. These genome sequences provide insights into the niche adaptation of these specific species in freshwater systems.

## GENOME ANNOUNCEMENT

Shewanella spp. are saprophytic, Gram-negative, rod-shaped members of the order Alteromonadales, family Alteromonadaceae, within the gamma subdivision of the Proteobacteria. They are frequently isolated from nonhuman sources, and some species (e.g., S. algae) are considered pathogenic in humans (1). The genus Shewanella is currently composed of more than 50 species that inhabit a range of aquatic environments (2, 3). The organisms potentially play key roles in environmental processes such as bioremediation of toxic elements and heavy metals (4). Also, they may serve as microbial fuel cells (5). Shewanella strains have also been isolated from the gastrointestinal tracts of freshwater fish species that live in environments that seasonally approach 0°C (6, 7). Here, we report the genome sequences of S. baltica strain CW2 and S. morhuae strain CW7, which were isolated as omega-3 fatty acid producers from the intestines of the freshwater fish Salvelinus namaycush and Coregonus clupeaformis, respectively.

PacBio sequencing with 100× coverage was performed as previously described for Shewanella sp. strain WE21 (6). Annotation was performed with the NCBI Prokaryotic Genome Annotation Pipeline (PGAP) (8). Additionally, the genomes were analyzed with the Rapid Annotations using Subsystems Technology (RAST) server (9). Specific genomic islands were recognized by MAUVE (10), secondary metabolism was analyzed by antiSMASH (11), clustered regularly interspaced short palindromic repeat (CRISPR) arrays were determined with CRISPRFinder (12), and prophage-related sequences were identified with PHASTER (13).

The final assembly for S. baltica strain CW2 resulted in one complete contig of 4,944,783 bp. The assembly of CW7 resulted in two large contigs totaling 4,303,233 bp. The assembly program may have failed to close the contigs due to a large repeat region at the ends. The GC contents of the genomes were 46.4 and 44%, respectively. Genome annotation for S. baltica resulted in 4,275 coding sequences (CDS) and 144 RNAs. S. morhuae had 3,764 CDSs and 137 RNAs.

In addition, both genomic sequences contained a type I secretion system (*lapBCDE*, *lapL, lapP*, and *rtx*); however, S. baltica strain CW2 had only one agglutination *rtx* toxin gene, and S. morhuae strain CW7 had 19 copies. Comparison of whole genomes by MAUVE with eight marine S. baltica strains available in the NCBI database showed that S. baltica strain CW2 had five genomic islands, ranging from 13.3 to 25.9 kb, that encode type I restriction-modification systems, hemoglobin-like proteins, a type II restriction-modification system, glutamate synthase, and anaerobic dimethyl sulfoxide reductase (chains A and B). Although related S. baltica marine isolates contain one to three plasmids (14), strain CW2 contains no plasmids or genes for a type III secretion system. Although the genes for OmpA were found in both strains, only the gene for the AquaporinZ protein was found in S. baltica, perhaps enabling it to grow in 0% NaCl (6). Finally, S. morhuae harbored a megaplasmid of 113.6 kb, which encodes a glutathione-dependent pathway of formaldehyde detoxification (*gfa*, *frmA*, *fghA*, *frmR*, and *regF*). No CRISPR arrays or prophage-like elements were found in either genomic sequence. Thus, these genomic sequences can facilitate future comprehensive comparisons and phylogenetic analyses of the niche adaptation of Shewanella communities.

### Accession number(s).

The complete genome sequence of S. baltica strain CW2 has been deposited at DDBJ/ENA/GenBank under the accession number CP028355. The version described in this paper is the first version, CP028355.1. The whole-genome shotgun project for S. morhuae strain CW7 has been deposited at DDBJ/ENA/GenBank under the accession number PYSG00000000. The version described in this paper is the first version, PYSG01000000.
